# Dark Matter of Primate Genomes: Satellite DNA Repeats and Their Evolutionary Dynamics

**DOI:** 10.3390/cells9122714

**Published:** 2020-12-18

**Authors:** Syed Farhan Ahmad, Worapong Singchat, Maryam Jehangir, Aorarat Suntronpong, Thitipong Panthum, Suchinda Malaivijitnond, Kornsorn Srikulnath

**Affiliations:** 1 Laboratory of Animal Cytogenetics and Comparative Genomics (ACCG), Department of Genetics, Faculty of Science, Kasetsart University, Bangkok 10900, Thailand; farhan.phd.unesp@gmail.com (S.F.A.); worapong.si@ku.th (W.S.); maryam.bioinfo.unesp@gmail.com (M.J.); aorarat.sun@ku.th (A.S.); thitipong.pa@ku.th (T.P.); 2 Special Research Unit for Wildlife Genomics (SRUWG), Department of Forest Biology, Faculty of Forestry, Kasetsart University, Bangkok 10900, Thailand; 3 Department of Structural and Functional Biology, Institute of Bioscience at Botucatu, São Paulo State University (UNESP), Botucatu, São Paulo 18618-689, Brazil; 4 National Primate Research Center of Thailand, Chulalongkorn University, Saraburi 18110, Thailand; suchinda.m@chula.ac.th; 5 Department of Biology, Faculty of Science, Chulalongkorn University, Bangkok 10330, Thailand; 6 Center of Excellence on Agricultural Biotechnology (AG-BIO/PERDO-CHE), Bangkok 10900, Thailand; 7 Omics Center for Agriculture, Bioresources, Food and Health, Kasetsart University (OmiKU), Bangkok 10900, Thailand

**Keywords:** non-human primates, alpha satellite, tandem repeats, heterochromatin, centromere, evolution

## Abstract

A substantial portion of the primate genome is composed of non-coding regions, so-called “dark matter”, which includes an abundance of tandemly repeated sequences called satellite DNA. Collectively known as the satellitome, this genomic component offers exciting evolutionary insights into aspects of primate genome biology that raise new questions and challenge existing paradigms. A complete human reference genome was recently reported with telomere-to-telomere human X chromosome assembly that resolved hundreds of dark regions, encompassing a 3.1 Mb centromeric satellite array that had not been identified previously. With the recent exponential increase in the availability of primate genomes, and the development of modern genomic and bioinformatics tools, extensive growth in our knowledge concerning the structure, function, and evolution of satellite elements is expected. The current state of knowledge on this topic is summarized, highlighting various types of primate-specific satellite repeats to compare their proportions across diverse lineages. Inter- and intraspecific variation of satellite repeats in the primate genome are reviewed. The functional significance of these sequences is discussed by describing how the transcriptional activity of satellite repeats can affect gene expression during different cellular processes. Sex-linked satellites are outlined, together with their respective genomic organization. Mechanisms are proposed whereby satellite repeats might have emerged as novel sequences during different evolutionary phases. Finally, the main challenges that hinder the detection of satellite DNA are outlined and an overview of the latest methodologies to address technological limitations is presented.

## 1. Introduction

The latest advances in genome sequencing technologies and an increase in the number of available genomes have presented novel opportunities for comparative and evolutionary genomics research. Increased knowledge regarding the patterns of primate genome contents and dynamics, such as great apes and macaques, has generated critical information regarding the evolutionary origin of the human genome and related biomedical predictions [1]. Assemblies of non-human primate (NHP) genomes have provided excellent resources to study genetic variations and similarities of model species used for biomedical research [2]. The first NHP genome to be sequenced and published was that of *Pan troglodytes* (chimpanzee) in 2005 [3], shortly followed by that of *Macaca mulatta* (rhesus macaque) [4]. These genome assemblies enabled investigation of the origin of human life through comparative genomics and evolutionary analysis to better understand the mechanisms of genetic changes that drive molecular evolution [2,3,4,5]. Two key issues in primate comparative genomics concern (i) understanding genomic association using cognitive science to elucidate the mechanisms of human diseases, and (ii) analysis of genomes to uncover the mechanisms of rapid human evolution [1]. Whole-genome sequences for genomes of more than 240 primate species (68% of the total) have now been assembled and are accessible online in the NCBI Assembly database (https://www.ncbi.nlm.nih.gov/assembly/?term=primate).

**Figure 1:**
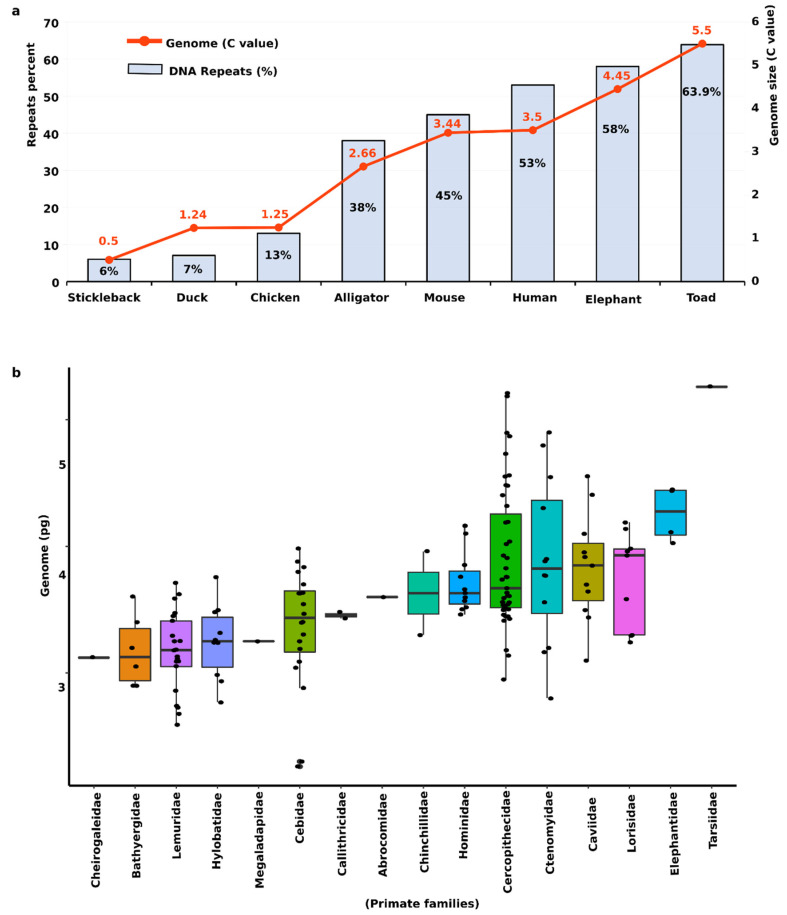
Overview of repeat contents in vertebrates and primate genome size variation. (**a**) Relationship of genome size (C value; red line) with repeat contents (blue bars) in representative vertebrate organisms. (**b**) Boxplot of the distribution and variability of genome size among primate families. Each dot represents a primate species. Note: Data are sourced from the Animal Genome Size Database (http://www.genomesize.com/) [14] and graphics were created in R.

Following successful completion of initial human genome sequencing, we now understand that in addition to the coding portion of the genome (a mere 1–2%), including protein-coding genes that instruct major development and functions, there is also a non-coding portion of the genome with astonishing implications of complexity [6]. The remaining 98–99% non-coding component of the genome comprises highly repetitive sequences and is usually underestimated in the assembled genomes; some parts may contain highly complex repetitive regions that remain undetected despite notable recent developments in sequencing technology. These are considered to be the “dark matter” of the genome [7]. After two decades of improvements, a complete and gapless telomere-to-telomere assembly of a human X chromosome was recently achieved [8]. This milestone has allowed the previous human reference genome (GRCh38) to be updated, and has resolved gaps within the dark matter spanning a novel centromeric satellite 3.1-Mb-long array that currently represents the most accurate and complete vertebrate genome produced to date. The non-coding portion forms the bulk of the genome with highly variable contents from one organism to another, thus making a substantial impact on the variation in genome size (Figure 1a). Such variation may be due to diverse abundances of repeated sequences including tandemly arranged repeats, transposable elements (TEs), and ribosomal genes [9,10,11,12,13]. Comparison of data from the Animal Genome Database [14] (http://www.genomesize.com/) shows substantial variation in the distribution of genome size among primate lineages, depending on repeat proportion (Figure 1b). Old World monkeys (family *Cercopithecidae*) and tarsiers (family *Tarsiidae)* have a comparatively larger genome than other lineages, which suggests that expansion of repeat sequences might have occurred during the evolution of their genomes (Figure 1b). Similar cases are observed in other amniote groups, such as mammals and reptiles [13]. Such variation is not correlated with the complexity of the organism. Variation in genome size among species may be associated with different proportions of repeat contents in their genomes [15,16,17] (Figure 1).

Primate genomes are enriched in repeats (more than 50%), some of which remain uncharacterized [18,19]. Similar to other vertebrates, primate genomes include an abundance of tandem repeats that are organized in such a pattern that the sequences are repeated directly adjacent to each other [20]. These repeat sequences consist of satellite DNA (satDNA), which is defined as tandemly arranged repeats that represent a considerable proportion of the heterochromatic portion of the eukaryotic genome, forming the main structural component (heterochromatin) of chromosomes [13,21,22,23,24,25,26]. SatDNA has been implicated in a variety of important functions, including segregation during cell division, homologous chromosomal pairing, kinetochore formation, chromatid attachment, chromosomal rearrangements, and differentiation of sex chromosomes [27,28,29,30,31,32,33]. Perhaps most importantly, satDNA can constitute rapidly evolving sequences of the genome [34,35,36] and is now considered to be important in driving genomic and karyotypic evolution [13,22,23,24,37].

**Figure 2:**
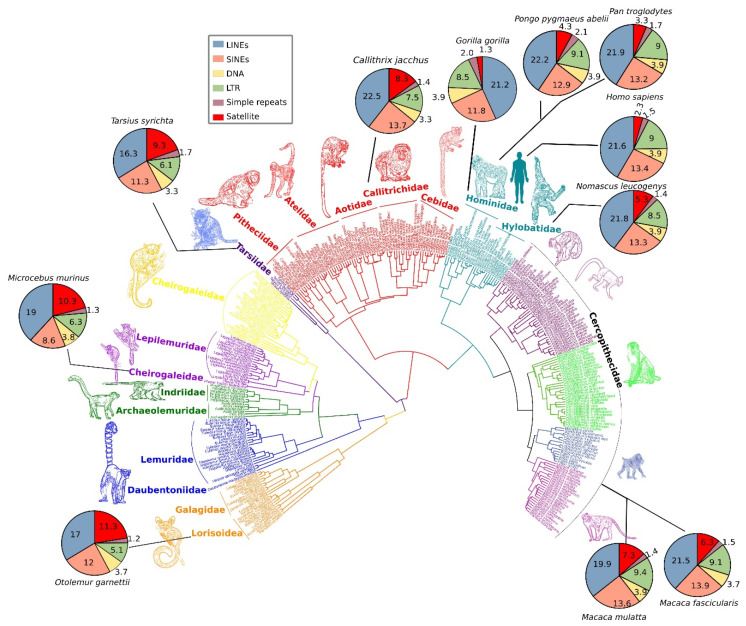
A comprehensive phylogeny of 301 primate species based on mitochondrial DNA sequences using Bayesian inference. Pie charts for selected common primate species show percentage differences of repeat types in the respective genomes. The abundance of satellite DNA in primate genomes varies considerably among lineages (red colored area of pie charts). Additionally, the comparative repeatomic landscape shows LINEs and SINEs emerged as the most expanded elements of primate genomes (blue- and orange-colored areas of pie charts) with consistent pattern across diverse lineages. Phylogenetic data were retrieved from the Primates Section of the 10kTrees website (https://10ktrees.nunn-lab.org/Primates/dataset.html) [53]. The phylogenetic tree was customized and filtered using iTOL v5 software (Interactive Tree Of Life; https://itol.embl.de/) [54]. Different colors represent different clades. Cartoons of the representative primates were drawn using Inkscape software.

In addition to satDNA, a substantial proportion of primate repeats is categorized as dispersed sequences. These sequences include TEs such as long interspersed nuclear elements (LINEs) and short interspersed nuclear elements (SINEs), which are the most abundant interspersed repeats in primate genomes (Figure 2) [38,39,40,41]. The evolutionary young (recently emerged) TEs, such as long terminal repeats (LTRs) and the retrotransposons SINE-VNTR-Alu (SVA), may play an important role in the regulation of gene expression and drive evolutionary divergence among primate species [42]. Insertion of new copies of TEs can accelerate the recombination rate; for example, Alu elements may increase the rate of unequal crossover [43].

The recent proliferation of new bioinformatics and computational tools has been particularly helpful for the assessment of the variation of repeats. Other developments include advanced next-generation sequencing (NGS) technologies and related software, and the increasing availability of online genomic databases [19,44], all of which allow more reliable testing of models of the functional aspects of the evolution of satDNA within the primate genome. Such analyses offer deep insights into the organization of satDNA within the primate genome and its possible roles in neutral and adaptive evolution. Although primate genomes have been utilized as resources to perform repeatomic analyses, with specific focus on transposable element (TE) contents [45], their satellitomes (complete sets of satDNA families in a genome) remain poorly understood, with just a few recent reports of novel satDNA families [46,47,48,49]. Currently available data on chromosomics and molecular and population genetics of satDNA in primates raise many hypotheses concerning their evolutionary origin, expansion in different genomic loci, and functional roles [33,46,47,48]. To shed further light on these phenomena, we have collected a range of evidence and propose the dynamics of satDNA in primates can be characterized as follows: (i) SatDNA repeats may follow an independent evolution in primate genomes and differences in their genomic abundance among taxa can increase with phylogenetic distance, (ii) the predominant satDNA families are conserved in primates with the exception of certain satDNA types that have undergone extreme divergence, (iii) specific portions of satDNA in the genome show population/species/lineage-level divergence and a paradoxical link with the evolution of centromeres, (iv) the Library model of satDNA evolution is still applicable in primate genome, and (v) satDNA transcriptional activity can mediate regulation of gene expressions that consequently influence wide ranging cellular phenomena. Here, we review and summarize the emerging discoveries of satDNA and discuss its impact on the reshaping of the evolution and dynamics of primate genomes. We highlight different types of primate satDNA and discuss their genomic organization, evolution, and function. We also present an overview of sex chromosomes linked to satDNA and provide detailed insight into lineage-specific divergence of these sequences. We describe an evolutionary model for satDNA and propose a mechanism for the main events that might have occurred during the genomic birth of satDNA. Current challenges in satDNA detection are also briefly discussed.

## 2. Satellite DNA Abundance in Different Primate Lineages

The genomes of most primates, such as monkeys, apes, and humans, comprise up to 50% repeat contents, of which satDNA may constitute as much as 10% of the total number of repeats [50,51]. RepeatMasker data [52] for different primate species indicate that their genomes can contain a highly variable proportion of satDNA (Figure 2). Comparison of these data shows that satellite repeats are highly abundant in certain families, such as nocturnal primates (superfamily *Lorisoidea*), strepsirrhine primates (family *Cheirogaleidae*), and haplorrhine primates (family *Tarsiidae*) (Figure 2), which suggests extensive expansion of satDNA in the genomes of these lineages. By contrast, in *Hominidae* and *Hylobatidae,* satellite repeats are comparatively low in abundance. The genomes of *Hominidae* and *Hylobatidae* are invaded by TEs at higher proportions compared with those of the *Lorisoidea, Cheirogaleidae,* and *Tarsiidae* lineages. This observation suggests that phylogenetically close lineages show similar patterns of satellite abundance in their genomes, whereas differences in abundance among taxa increase with phylogenetic distance. The phylogenetic tree of Figure 2 was retrieved from 10kTrees website [53] and designed using Interactive Tree of Life, iTOL [54]. However, data on the relative percentage of satDNA in different primate genomes must be treated carefully, and precise information on satDNA abundance in primate genomes is still lacking due to misassemblies, gaps, and unresolved assembled centromeric regions that span these repeats [19,55].

SatDNAs were initially identified by their buoyant densities (in g/mL) on cesium chloride gradients [56]. This technique was formerly employed for satDNA detection and biased procedures. This technique can identify a single satellite or sometimes multiple satellites in a genome but cannot detect the entire set of satellite families. Modern techniques, such as NGS and fluorescence in situ hybridization (FISH), have replaced traditional methods and have substantially improved detection and characterization of satDNA [19]. This methodological shift has brought advances in the identification of different repeat types and structural units of satDNA in primate genomes [41,47]. Using cytogenetics, the genomic organization and diversity of satDNA have been widely studied mostly in humans, and to some extent in other primate genomes. As a result, a wealth of knowledge is now available on the localizations of satDNA repeats, their lengths, and different units, variability, and number of copies in different genomes [55,57,58,59,60,61,62,63]. These repeats can be categorized as different types of satDNA to better understand their roles, evolution, and applications in phylogenetic analyses. This can include satellites that are generally shared across all eukaryotic lineages and those that are exclusive to primate genomes.

## 3. General and Primate-Specific satDNA Types

Certain tandem repeat sequences can be classified by the number of base pairs (bp) into two types as microsatellites (ranging in length from one to six or more bp) and minisatellites (usually from 10 to 100 bp) [64]. The human genome contains as much as 3% microsatellites [65] and several thousand chromosomal loci enriched with minisatellites [66], also called variable number tandem repeats (VNTRs) [67]. Previous isolation of microsatellites from the human genome has enabled researchers to amplify these sequences in several NHP species, including apes, baboons, macaques, and some platyrrhine monkeys [68,69,70,71,72,73,74]. Microsatellites tend to accumulate many substitutions and/or insertions/deletions, and are thus considered to show limited conservation across primate lineages [75]. Many conserved microsatellites, such as AP74, which was discovered in New World monkeys, exhibit similar sequence length (up to 176 bp) in monkeys and humans [76,77]. Boán et al. [78] identified the minisatellite MsH42 in the human genome and performed a comparative analysis in 11 NHP species. Phylogenetic analysis detected several variants of MsH42 and the evolutionary birth of minisatellites in the primate genome was hypothesized. According to this hypothesis, the evolutionary birth of MsH42 took place within an intron early in primate lineage evolution and more than 40 million years ago. Then, various mutations including insertions, duplications, and single nucleotide polymorphism of repeat blocks were probably the major forces governing the generation of this minisatellite and its divergence throughout primate evolution [78]. Certain (TTAGGG)_n_ sequences, which are specific monomers of microsatellites, can be repeated multiple times, eventually forming the bulk of the telomeric region up to 15 kb on human chromosomes [79,80]. These telomeric repeats can serve as binding sites for certain nucleoproteins, such as TRF1, TRF2, and POT1, forming a complex termed “shelterin” [81] that interacts with a ribonucleoprotein [82]. This complex is involved in DNA repair processes and the protection against degradation of chromosomal ends [83].

Well-characterized telomeric satellites of the human genome can also be applied broadly as informative markers to study a variety of hominoid species owing to multiallelic variation and a high degree of heterozygosity [70]. The MsH42 locus shows high similarity with immunoglobulin regions and is involved in recombination events as well as in promoting high rates of unequal crossovers [78,84,85]. The telomeres harbor short stretches of sequences termed interstitial telomeric sequences (ITSs), which are located far from the chromosomal ends. To trace the evolutionary origin of these sequences in NHP genomes, 22 ITS loci from the human genome were compared with their orthologs in 12 NHPs, representing species such as great apes, gibbons, Old World monkeys, and New World monkeys. Comparison of sequences indicated that, unlike other microsatellites, these ITS sequences were not derived from expansion of pre-existing TTAGGG monomers but rather emerged abruptly during genome evolution in primates as a result of double-strand break repair [86]. Similar findings were observed from investigation of a chimpanzee-specific ITS. A universal satDNA classification is still the subject of debate; however, most commonly, satDNA can be grouped according to position and association with different chromosomal loci. SatDNA is primarily clustered within the heterochromatin regions of primate chromosomes. The heterochromatic portion is mainly localized in centromeric and telomeric regions, and sometimes within the interstitial regions of the chromosomes [87], whereas satDNA sequences are mostly located in centromeric regions, and the nearby pericentromeres may be enriched with TEs. Different types of primate satDNA are discussed and summarized as Supplementary Table S1.

### 3.1. Centromeric and Pericentromeric satDNA: Primate-Specific Alpha Satellites and HORS

**Figure 3:**
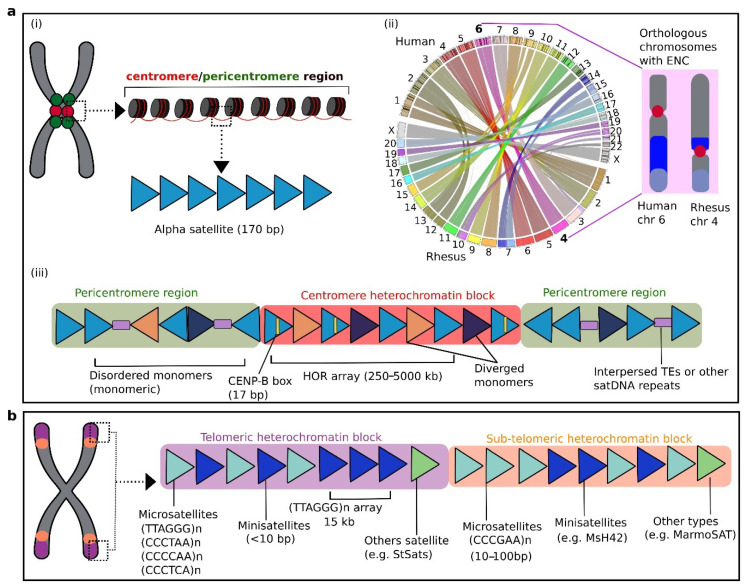
Schematic illustration of satellite DNA repeats and their organization in primate genomes. (**a**) (i) Primate centromeric (red) and pericentromeric (green) regions are enriched with alpha satellite (AS) DNA as the most abundant satellite repeats of primate genomes and form the bulk of the heterochromatin core. (ii) A sketch highlighting the orthologous chromosomes and centromeric repositioning as evolutionary new centromeres (ENCs) between human and rhesus macaque. The circos plot depicts the syntenic relationship between the two genomes. Circos graphics was plotted using Synteny Portal [117]. Note that human chromosome 6 is completely orthologous to macaque chromosome 4, with evolved centromeres [109,116]. (iii) The AS constitute the tandem repeat units (blue triangles) and can be either organized as disordered arrays (monomeric) mostly located in pericentromeres, or highly ordered in a head-to-tail fashion (HORs) forming longer arrays in centromeres. Some monomers may also have a short sequence termed the CENP-B box (yellow line), which binds the centromeric regions to the DNA-binding proteins. Diverged monomers (orange and dark triangles), and interspersed repeats (purple rectangles) are also depicted. (**b**) Telomeric and subtelomeric regions of primate chromosomes are enriched with distinct microsatellites (light blue) and minisatellites (dark blue). Various primate-associated satellite examples are shown.

The centromere cores of human chromosomes span abundant and highly enriched stretches of satDNA, and are surrounded by heterochromatin containing a combination of short satDNA sequences and retroelements [29,88]. Occasionally, these centromeric regions are termed “satellite centromeres” [89]. The centromere is an important region of the chromosome for preservation of genetic materials and plays a critical role in chromosome segregation, cell division, kinetochore organization, and spindle attachment [89,90,91,92]. In primates, the bulk of the centromere is composed of the pancentromeric alpha satellite (AS), organized as stretches of 171 bp monomers in a head-to-tail fashion extending for ~250 kbp up to ~5 Mbp per chromosome [93,94,95,96] (Figure 3a(i)). This structure has been reported across diverse groups, including great apes, Old World monkeys, and New World monkeys [96,97,98,99,100,101,102]. These centromere-associated satellites are arranged as superfamilies (SFs) that can be orthologous between human and gorilla [60]. The surrounding pericentromeric satDNA are essential elements that assist in stabilization of DNA–protein binding and regulation of chromosome segregation [58,61]. These pericentromeric satellites vary greatly across NHP species but can be conserved among closely related species or may be species-specific [20,103]. For instance, a large block of human chromosome 9 that spans a pericentromeric area enriched with satellite III (SatIII) shares close homology with the gorilla sequence [104]. The Y chromosome of NHPs may carry higher numbers of copies of satellite III sequences than the human Y chromosome [105]. FISH mapping of the pericentromeric-type satellite pW-1 SatIII DNA on chromosomes of various NHP species showed that these sequences might be lacking in the genomes of squirrel monkey (*Saimiri sciureus*) and baboon (*Papio hamadryas*) [105]. These centromeric satellites can vary substantially across different species, but certain species-specific or even highly conserved satDNA may also be present in the centromere domains [20,103]. For example, two major families of centromeric satellites, termed C1 and C2, detected in Old World monkey species crested mona monkey (*Cercopithecus pogonias)* and sun-tailed monkey (*Cercopithecus solatus*) have remained highly conserved [48]. For Old World monkeys, apes, and humans, each genome harbors evolutionarily distinct AS monomers [106]. Although most primate centromeres can be enriched with satellites repeats, there are certain chromosomes of orangutan that comprise non-repeated centromeres [92,107,108,109,110]. In such cases, the centromeres may resemble newly formed neocentromeres as a result of disruption in the centromeric region, such as in humans [92,111]. Such non-repeated centromeres are likely to be evolutionary new centromeres (ENCs), forming neocentromeres that might have subsequently gained repeat sequences to stabilize the genome and become fixed in populations. This phenomenon can also occur in the centromeres of several non-primate species, such as horse and chicken [112,113]. In the following, we focus mainly on the predominant centromeric satDNA in primate genomes as AS repeats.

The AS repeats were first observed as tandem repeats in the African green monkey (*Chlorocebus aethiops*) genome [93], followed by identification of homologous repeats in New World monkeys and apes [96,114]. These sequences are considered to be critical components for the various functions of primate centromeres [94]. Previous results suggest that AS sequences were involved in stabilization of ENCs after their emergence in primate genomes [109,115]. Human and macaque chromosomes contain a total of 14 ENCs, of which nine ENCs in the macaque genome show abundant arrays of AS [109]. Interestingly, ENCs occur in macaque chromosome 4 and human chromosome 6, which are orthologous to each other (Figure 3a(ii)) [109,116,117].

The AS monomer size is 171 bp, tandemly arranged in a head-to-tail manner, and shows as much as 70% sequence similarity. The combined monomers can form a long array spanning an uninterrupted 250–5000 kb stretch of repeated satellites, giving rise to high-order repeats (HORs) (Figure 3a(iii)). A certain monomer in the HORs with a sequence size of 17 bp is termed the CENP-B box. This motif acts as a protein-binding site for a centromeric CENP-B protein in primates. The human genome project, which was declared complete in 2003, was still unable to recover a large proportion of the centromeric and other repeats, including more than 10% of the contents of the whole genome, mainly sex chromosomes. However, subsequent technological developments enabled assembly of the entire human Y chromosomal centromere [62,118]. The Y chromosome assembly could be used as a reference sequence to extend evolutionary insights into the centromeric repeats of NHPs for which Y chromosome assemblies have not been hitherto accomplished.

In primates, the flanked regions of centromeres have specialized HORs arrays, whereas AS sequences are organized as non-structured and heterogeneous repeats, forming distinctive pericentromeres. In these pericentromeres, AS sequence repeats are arranged as monomers instead of HORs and are interrupted with additional elements, mainly retrotranposable elements in humans [119] (Figure 3a(iii)), which may also be common to other primate genomes. The pericentromeres of certain human chromosomes may also show enrichment of several other repeat sequences, including the 5 bp satDNA II and III type sequences [103,120]. The AS sequences can show nucleotide variation when one monomer is compared with the repeats of the same array, with nucleotide identity ranging from 70% to 90%. The sequences of a monomer in one array may show up to 95% similarity with its counterpart unit in the other array at the same locus [63,121,122]. In the human genome, the organization of HORs with their monomer units has been extensively studied [65,97,123,124], and shows the occurrence of various subfamilies of chromosome-specific AS sequences. The sequences of HORs in great apes, such as orangutan, gorilla, and chimpanzee, show a lower degree of variation in comparison with HORs observed in the human genome [125,126,127,128]. Initially, it was presumed that the organization of HORs might be restricted to hominids; however, HORs were subsequently detected in the genomes of gibbons [101,102,129] and of Old World and New World monkeys [48,102,130]. During the evolution of the primate genome, the 170 bp AS monomer underwent a series of sequence variations [87]. A novel AS monomer type of 189 bp was discovered in the centromeres of gorilla [60]. Chromosome-specific subfamilies are absent in Old World and New World monkeys as well as in gibbons [87,101,106]. Cloning, sequencing, and hybridization of acrocentric chromosomes revealed novel AS sequence repeats in Azara’s owl monkey (*Aotus azarae*), which is a species of New World monkey [22,23]. These repeats include three megasatellites, namely OwlRep, OwlAlp1, and OwlAlp2, which vary in size from 184 to 344 bp as identified in the centromeric and pericentromeric regions. Analysis of retina samples using three-dimensional FISH revealed that OwlRep is the major component of heterochromatin, which indicates its role in the evolution of night vision in this species [131,132]. Recently, Cacheux et al. [49] investigated the evolutionary dynamics of AS sequence repeats and their diversity in the Old World monkeys *Cercopithecus pogonias* and *C. solatus* using targeted sequencing and FISH mapping. These authors reported evidence of chromosome-specific subfamilies that might have evolved through homogenization. The OwlRep repeat shows ~82% homology with a satellite sequence termed HSAT6, which is a 126 bp long tandem centromeric repeat. The HSAT6 sequence was also detected in the owl monkey genome, and comparative analysis revealed its broad distribution among hominoids and New World and Old World monkeys. Phylogenetic analysis confirmed that OwlRep evolved from HSAT6 [132].

In addition to AS, an additional type of satellite family termed the beta satellite is distributed in the heterochromatin of primates [133,134,135]. Beta satDNA are repeats that comprise ~68 bp monomers. They are predominantly organized in the shorter arm of acrocentric chromosomes and arranged in stretches several kb in length [136,137,138,139]. The beta satDNA repeats can form complexes with arrays of specific repeats, termed D4Z4 repeats, at certain acrocentric loci, such as 10q26 and 4q35 [140,141]. Evolutionary analyses involving cloning and FISH experiments have predicted that 4q35 containing D4Z4 repeats might represent an ancestral locus with an extensively radiated sequence region that evolved after the divergence of hominoids and Old World monkeys [142,143,144]. The origin and evolution of beta satDNA vary in diverse species of hominids, such as humans, chimpanzee, and gorilla [145,146]. FISH mapping data confirm that D4Z4 is also conserved in Old World and New World monkeys, whereas in primates distantly related to humans (e.g., lemurs), this sequence has retained tandem repetition but conservation is limited to promotor regions [147]. Genomic analysis of orangutan has revealed the origin of beta satDNA in earlier ancestors of hominoids and shows that these repeats are preferentially located in pericentromeres [135]. This study concluded that these repeats originated as low copies, remained non-duplicated in the early ape ancestors, and later evolved as duplicons acquiring the typical characteristics of classical satellites in humans and other primates. Adjacent to ASs, the classical non-alphoid satDNA repeat families I, II, and III are located in pericentromeres of human chromosomes [95]. The human genome includes the Sat III family, which is composed of GGAAT and GGAGT repeat sequences in different percentages. The satellite III family is mainly localized on the short arm of acrocentric chromosomes in humans and other primate species. This family is also present in the chimpanzee, gorilla, and orangutan genomes [148,149]. The chromosomal organization of this satellite family has provided interesting evolutionary insights into primate genomes [105]. Sequence comparisons have detected variation across different primate species and suggest that the Sat III family might have appeared ~16–23 million years ago in Hominoidea [105]. The evolutionary origin and extensive diversification of centromeric satellites in primate genomes remain unclear; however, it is speculated that TEs are the possible progenitors and sources that form novel satellites by insertions into existing satellite regions [119].

### 3.2. Telomeric and Subtelomeric satDNA

The telomere is located at the end of the chromosome and is enriched with a non-coding, repetitive DNA sequence. The 500 kb region of each chromosomal arm terminal is the so-called subtelomeric region [150]. Both telomere and subtelomere have high-density of satDNA repeats. Telomeric regions of the primate genome show a high frequency of minisatellites, which also occur in other loci of chromosomes [67,151]. The bulk of telomeric-specific regions are mainly composed of (TTAGGG)_n_ microsatellites in humans [79]. Adjacent to the telomere, the subtelomere region is mostly enriched in rapidly evolving satellite repeats with variable levels of repetitiveness and size [57,152,153]. Although these subtelomeric satellites can be species-specific and often chromosome-specific, there are also satellites that remain highly conserved [154]. The microsatellites (CCCTAA)_n_, (CCCCAA)_n_, and (CCCTCA)_n_ are present in telomeres of primates [155], whereas (CCCGAA)_n_ is restricted to subtelomeres [156] (Figure 3b). In New World monkeys, the subtelomeres can carry novel satDNA sequences. The subtelomeric regions of callitrichid monkeys harbor a satellite termed MarmoSAT that is composed of a 171 bp motif [157]. The MarmoSAT occurs as a monomer, whereas in common marmoset (*Callithrix jacchus*) it is organized in HORs with a sequence of 338 bp. Recently, some intriguing groups of satDNA sequences enriched with AT nucleotides, termed StSats, have been reported in telomeres of humans and great apes, including bonobo, chimpanzee, gorilla, and orangutan [47]. The StSats are located in proximity to telomeric regions [158,159,160]. Astonishingly, these satellites are very highly enriched in the gorilla and chimpanzee genomes compared with their abundance in humans [47]. Previously, it was hypothesized that these repeats occurred in hominid ancestors and were lost in humans [158,159,160]. The abundance of StSats repeats in the bonobo, chimpanzee, and gorilla genomes indicates that these sequences might contribute to important genomic functions in these species. Different functions have been proposed for these repeats that include their role in meiosis, telomere clustering, and control of replication duration with telomeric regions [158,159,160].

The distribution of two distinct satellite repeats, termed Cap-A and Cap-B, was reported in a New World monkey species, *Cebus paella* [161]. The Cap-A sequence is 1500 bp long and forms heterochromatic blocks in the interstitial sites of chromosome 11 and a few telomeric regions. This suggests that this sequence underwent a new episode of amplification in New World monkeys. This satDNA repeat is absent in most marmoset species and present in species of the family *Cebidae*. By contrast, the Cap-B satellite, which is 342 bp long, is mainly localized in the centromeric regions of many chromosomes of New World monkeys. The Cap-B monomer sequence shares more than 60% identity with AS repeats, which indicates that Cap-B might be the New World monkey homolog of Old World monkey AS repeat sequences. Telomeric satDNA sequences can participate in the formation and maintenance of telomeres, and may have an incidental role in cases losing of conventional telomeric repeats. In this way, telomeric ends are stabilized by satDNA [162]. Further, it has been demonstrated that telomere-like sequences interspersed within subtelomeric DNA may also play a role in subtelomeric recombination and transcription, via alternative lengthening of the telomere pathway and in telomere healing [163]. It is necessary to identify and characterize the telomeric/centromeric satDNA sequences particularly at the breakpoint sites because of their role in mediating chromosomal rearrangements [164] that occurred during primate evolution. Such analyses have been performed with regard to the gorilla-specific translocation [165] as well as the chromosome scale variations that serve to distinguish human and chimpanzee chromosomes. Various hotspot rearrangement regions of the gibbon genome have also been characterized [166]. In contrast to the great apes, gibbons have chromosomes with higher levels of rearrangement compared to ancestral primate karyotypes [167]. A comparison of human and chimpanzee karyotypes showed that two ancestral chromosomal homologs of chimpanzee chromosomes 12 and 13 underwent a fusion event to give rise to human chromosome 2 [168]. This fusion was mediated by recombination between telomeric satDNA repeats of the two sub-metacentric ancestral chromosomes. The hyper-expanded repeats are localized in subtelomeric regions of chimpanzee chromosomes. These repeat enriched regions are also prone to other types of rearrangement events such as duplicative transpositions and inter-chromosomal sequence variations [169]. Since many primate-specific rearranged loci are enriched with high-copy repetitive sequence elements such as alpha satDNA repeats, SINEs, LINEs, and LTRs, a range of different molecular mechanisms were probably involved in promoting chromosomal breakage during the evolution of primate genomes [164]. Genome-wide scale analyses at higher resolution are necessary to determine the precise mechanisms underlying the different types of rearrangement, and to assess their relative contribution to the process of evolutionary change.

## 4. Sex Chromosomes: A High-Impact Arena for satDNA

In addition to the distribution of satDNA repeats on autosomes, these sequences are specifically enriched in distinct loci of particular chromosomes such as microchromosomes, supernumerary chromosomes, and sex chromosomes [13,170,171]. In primates, highly heteromorphic sex chromosomes have evolved from a pair of autosomes [172]. Divergence and erosion of the Y chromosome resulted in loss of several functional genes and accumulation of different repeats, including TEs and satellites [173,174,175,176,177]. FISH mapping of the SatIII family in different primate species revealed that these satellite repeats occur on the Y chromosome of humans, chimpanzee, gorilla, orangutan, and gibbons [105]. Weak hybridization signals on the human Y chromosome indicate fewer copies of these repeats than in other primate species. FISH mapping of a subtelomeric satellite, MarmoSAT, in four species of marmosets confirmed the occurrence of these sequences on both X and Y chromosomes [157]. MarmoSAT is localized to the short arm, whereas the telomeric repeats (TTAGGG)_4_ are distributed on the long arms of the sex chromosomes of *Callithrix penicillata* and *C. geoffroyi.* RNA-sequencing analysis further revealed that MarmoSAT is transcriptionally active with higher levels of expression in spleen, thymus, and heart tissues, which suggests it may play a role in telomeric chromatin [157]. An additional sex-linked satDNA is gamma satellite, which comprises 220 bp repeated GC-enriched units and is mostly embedded with AS sequences to form repetitive clusters [178,179,180]. Gamma satellite is mainly localized in pericentromeres of the X and Y chromosomes in humans [28,179]. An interesting satellite of the human Y chromosome is HSAT 1, which occurs in the heterochromatin [181]. This HSAT 1 is surrounded by palindromic AT-rich repeats and an AluSc element. Together, these three units form a sequence with a tripartite structure. These repeats are also localized on the autosomes but not on the sex chromosomes of NHPs. Exclusive occurrence on the human Y chromosome shows that this sequence has been duplicated on the Y chromosome after divergence of the human genome from its common ancestor [181]. Characterization of the diversity of repeat sequences has resulted in the detection of AS sequence subfamilies on the Y chromosome of the Old World monkey *C. solatus* [48]. The C3 and C4 satellites are localized on the Y chromosome with high copy numbers but are almost absent in autosomes. The Y-specific organization of these satellite dimers (C3 and C4) emphasizes that the Y chromosome does not undergo recombination. The Y-linked satellite repeats in many primate species have remained unexplored, except in gorilla, chimpanzee, and humans, for which Y chromosomes have been almost completely assembled [176,182,183].

An advanced methodology was proposed to identify Y-linked sequences in humans [184]. This technique located 119 scaffolds of ~18 Mb present on the human Y chromosome. A total of 34 sequences of the 119 scaffolds constitute a higher percentage (74%) of repeats, mainly including Y-linked satellites. These satellites map to the centromere and Yq12 band of the Y chromosome. Recently, short- and long-read sequencing analysis has uncovered male-biased satellites among great ape species [47]. Differences in density between male and female repeats resulted in identification of 18 satDNA sequences that included the human Y-specific “(AATGG)_n_” sequence with its three types of satellite units: DYZ1, DYZ17, and DYZ18 [165]. These three satellites have also been mapped on the Y chromosome of bonobo, chimpanzee, gorilla, and orangutan [105]. Different StSats sequences have been identified on the Y chromosome of bonobo and gorilla [47]. These studies have greatly improved current knowledge of satellite contents of the Y chromosome in apes. Differences in abundance or enrichment of unique satellite repeats were previously considered to be a primary step in distinguishing the X and Y sex chromosomes [185]. An emerging hypothesis states that the composition of Y heterochromatin may differ from the remaining chromosomes owing to several factors that include lack of Y recombination, the putative role of heterochromatin in silencing of Y, and the smaller effective population size of Y [175,186,187]. Apart from the Y chromosome, the human X chromosome is among the best studied sex chromosomes among primates. FISH mapping revealed the abundance of a type of HORs termed DXZ1, which is located in the functional centromere of the X chromosome [29]. The accumulation of HORs in the X and Y chromosomes has been linked to topoisomerase II cleavage activity detected in active centromeric regions [188,189,190]. Functional and genomic analysis has further unveiled many interesting findings on the pericentromere of the human X chromosome, which is why the X chromosome has emerged as an exciting model system for investigation of the centromere [29]. The particular occurrence of DXZ1 HORs proximal to the shorter arm of the X chromosome is especially important to gain advanced knowledge of the function of the centromere. A survey of satellite repeats in several primate species compared sequences in the pericentromeric region of the X chromosome and offered new insights on the evolution of the centromere [30]. The evolutionary analyses showed the addition of new sequences into centromeric regions as a series of punctuated events including frequent homogenization and inter-chromosomal exchanges of monomeric AS monomers in early primates. Phylogenetic analysis of X-linked AS monomers detected certain domains containing recently evolved LINE retrotransposons flanked with these satellites. In-depth evolutionary comparison of this junction revealed a striking conservation of AS sequences, which supports its ancestral nature in primates such as baboon, chimpanzee, gorilla, orangutan, macaque, and vervet monkey. In addition, this comparative analysis demonstrated that the centromere of the X chromosome in primates might have evolved as a result of expansion events of repeats. Among the most studied X-linked satellites is the macrosatellite, DXZ4, which consists of 3 kb repetitive units at the Xq23 position [191]. FISH mapping has revealed the organization of DXZ4 on the X chromosomes of chimpanzee, gorilla, and orangutan [192]. These sequences were subsequently mapped on the X chromosomes of male and female rhesus macaque *Macaca mulatta* [48]. Southern blot analysis revealed hybridized fragments of different sizes (50–350 kb), which points to its possible VNTR nature in primates, including great apes and Old World and New World monkeys [147]. Several important features, including the promotor, CpG sites, GC content, and CTCF binding site associated with the DXZ4 satellite have been explored in phylogenetically distant primate species. Integrative genomic approaches, coupled with chromatin conformation (immunoprecipitation and immunofluorescence) experiments in macaques, further indicate that DXZ4 is organized in heterochromatin on the active X chromosome, whereas this sequence is packaged in euchromatin on the inactive X chromosome [147].

## 5. Transcription of Satellite Repeats: Hidden Switches for Dialing Gene Expression Up and Down

**Figure 4:**
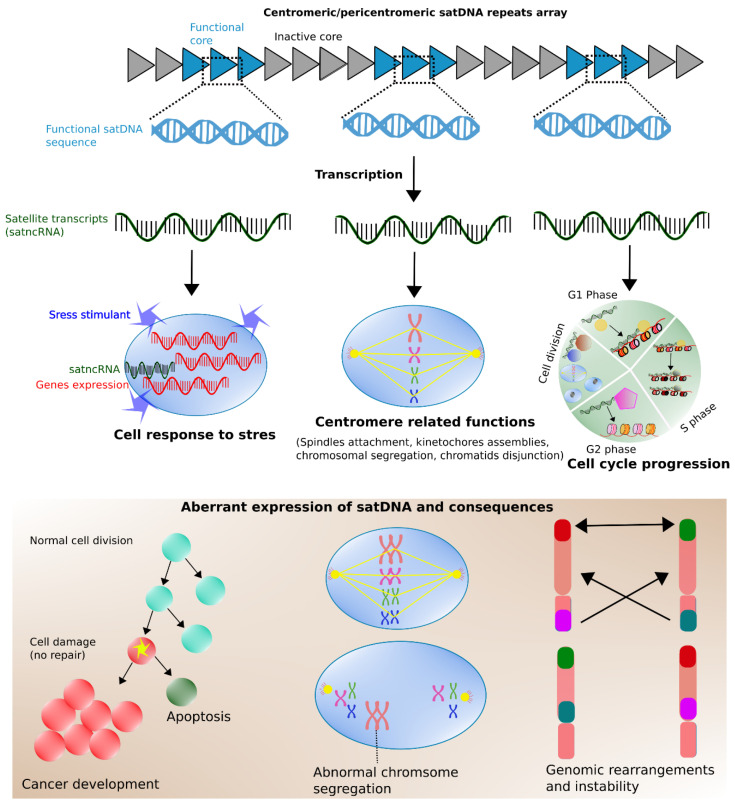
Diagrammatic summary of satellite DNA transcription highlighting various functional roles in cellular processes. The most studied transcripts of satellite repeats are those localized in the centromeres. The centromere/pericentromere core contains the active regions with satellite sequences that can be transcribed into satncRNA. These satncRNAs are associated with various functions. For example, during cellular stress, the satncRNA can regulate the expression of important genes, such as HSF1 (Heat Shock Factor 1), to produce nuclear stress bodies. In addition, satncRNA can also regulate the splicing of associated genes that are vital in stress responses. More importantly, satncRNA transcripts have been linked with centromere-related functions and cell-cycle progression. During the G1 phase, the satncRNA can facilitate the loading of CENP-A (yellow circle) at centromeres, which is distributed to every daughter strand in the S phase. In the G2 phase, the satncRNA transcripts form associations with SUV39H1 (purple pentagon) before initiation of cell division. During mitosis, satncRNA binds with SGO1 and AURORA B proteins, and assists in kinetochore assembly, spindle attachment, and chromosome segregation-related functions.

Satellite repeats have long been regarded as junk DNA [193]. However, there is increasing evidence to suggest the functional importance of these sequences. The transcriptional activity of satellites is a well-known feature in diverse species [194]. SatDNA sequences have been determined to be involved in various functions, such as developmental processes, stress response, cell proliferation, and cancer [195] (Figure 4). In principle, satellite transcripts are most likely associated with important genome functions, for example, centromere structure, kinetochore assemblies, and chromosomal segregation [58,87,195]. Recently, applications involving new methods of sequencing and bioinformatic analysis have enabled investigation of the functioning of satDNA in primates [196]. Alpha satellite non-coding RNA (satncRNA) has been detected in a prenucleosomal complex that contains CENP-A and HJURP [197]. During the G1 phase, RNA polymerase II associates with chromatin fibers when CENP-A and HJURP bind with a 1.3 kb satncRNA, which assists in localization of CENPA-A on centromeric chromatin. The absence of satncRNA has been linked with several mitotic defects and decreased levels of CENP-A recruitment, thus emphasizing its important role in centromere-related functions. In addition, in human cells satncRNA has been associated with heterochromatin formation by recruiting an enzyme termed SUV39H that can bind with the pericentromere [198]. Other crucial functions of AS repeats include regulation of spindle attachments, maintenance of heterochromatin, and disjunction of chromatids (Figure 4) [198,199,200,201,202]. These phenomena might be regulated by the association of satncRNA transcripts with AURORA B and SGO1 proteins [199], which might further associate with SUV39H1 proteins and promote heterochromatin stability [198,200,202]. It was previously believed that the heterochromatic portion of chromosomal regions are transcriptionally silent; however, satellite sequences in centromeres are actively transcribed during cell division [203,204], which plays an important role in kinetochore preservation and centromere cohesion [205,206]. The satDNA transcripts can perform different key functions that appear to be linked with their chromosomal locus. Transcripts of pericentromeric satellites are involved in the formation of chromatin. The SatIII transcripts can play an important role during the cellular response to stress in humans. In particular, heat shock factors [207,208] can initiate SatIII transcription and form nuclear stress bodies surrounding SatIII loci [209]. This can further cause splicing of stress response-associated genes, subsequently downregulating their transcription and preventing stress-induced apoptosis and subsequent cell death [210]. The SatIII transcripts are not only associated specifically with thermal stress response, and have been detected in the absence of stress [211]. Other functions of satDNA transcripts are kinetochore assembly, regulation of telomere capping and elongation, epigenetic control of heterochromatic regions, and gene expression [58,205,212].

The genomes of great apes contain three different types of AS monomers such as (AATGG)_n_, (TTAGGG)_n_, and AT-rich 32-monomer satellites. These sequences are present in abundance and are particularly interesting because of their function in great apes. The (AATGG)_n_ satellite, which is the source of the human HSAT2 and HSAT3 satellites, is transcribed into a long non-coding RNA that is important in thermal stress response [210]. This satellite also occurs in orangutan but its variability remains unknown in many ape species. The satellite (TTAGGG)_n_ localized in the telomere is critical in aging, cell division, and genome stability [213,214]. Another subterminal satellite (StSat) repeat, consisting of 32-bp-long AT-rich units, occurs in the proximity of subtelomeric regions in bonobo, chimpanzee, and gorilla, and is involved in telomere metabolism [158,160,215,216,217,218]. An irregular StSat expression level has been increasingly linked with cancer development [195]. The overexpression of satellites during stress can be analogous to cancer because certain features, such as abnormal chromosomal segregation, aneuploidy, or reduction of chromatid cohesion, prevail in both states. Hypomethylated pericentromeric DNA and up-regulation of satellite transcripts have been reported in cancer [219,220]. The highly repetitive nature, duplicated copies, and multiple genomic positions make transcriptional analysis of satDNA sequences a challenging task. The commonly available short-read NGS technologies show substantial limitations for investigation of satellite transcripts and their expression dynamics. A major shortcoming of short-read sequences is their inefficiency for assembly of the transcripts of large repeats. The recent advent of ultra-long-read sequencing may overcome this limitation in the near future. Transcriptional analysis of satellite repeats, under normal cellular functioning and in response to disease infection, has become an intriguing focus of ongoing research. However, much remains unclear about the expression of these sequences, and methodological improvements are needed to attain an improved understanding of their transcript functions.

## 6. Species and Population-Specific Variation: An Auspicious satDNA Feature for Genome Evolution

The evolution of satellite repeats in primates is extremely complex because some have remained conserved throughout evolution while others exhibit dynamic variation within the same population [221]. Heterochromatic sequences show remarkable interspecific variability in structure and size. This variation has also been detected between phylogenetically close primate species. The long arm of the human and gorilla Y chromosome carries heterochromatin as a major component, whereas heterochromatin is almost absent in chimpanzee [222]. Intraspecific variation in heterochromatin across human populations have been investigated intensively [62,223]. A Y-linked satellite of size 3.6 kb, namely HSAT3 in humans, contains a DYZ1 sequence that shows substantial variation among individuals and populations [223]. Similarly, centromere repeats of human X chromosomes are highly variable in sequence length among populations [62]. Certain human populations have shorter (15 kb) arrays within the heterochromatin of the neocentromeres that can result in defective sister-chromatid cohesion [224].

**Figure 5:**
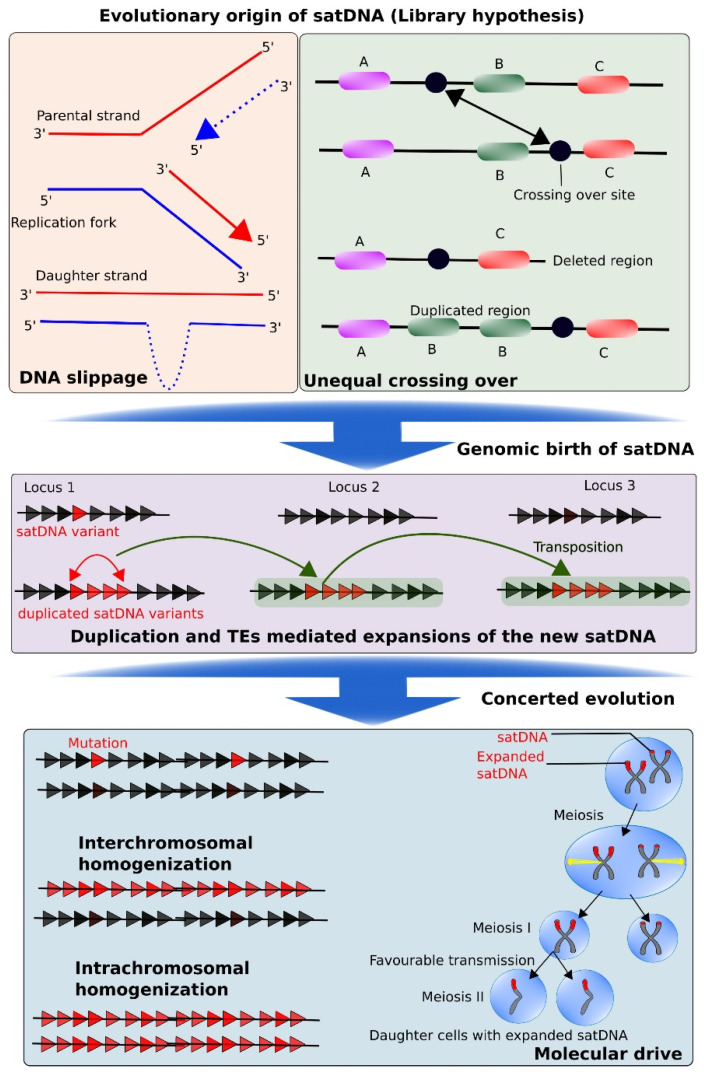
Model of satellite DNA evolution. Genomic birth of satellite DNA can occur as a result of different mechanisms. According to the Library hypothesis, the two main proposed phenomena are DNA replication slippage and unequal crossing over, which can cause mutations and de novo formation of satellite DNA (variants shown as red triangles). The newly formed satellite region can undergo several duplications and subsequent transposition events that expand the new satellite throughout the genome. Transposable elements mediate the spreading of newly evolved satellite repeats to different loci. This is followed by cohesive evolution of the genomic region to homogenize the entire array through selection. Finally, the evolved satellite repeats are established by sexual reproduction. The chromosome region with expanded satellite is inherited preferentially via a molecular mechanism known as “drive”. Two homologs have the same satellite DNA (red) but with a larger centromere, and one homolog with expanded satellites is attracted by spindle fibers and driven to the daughter cells.

Sequence composition analysis of the SatIII DNA subfamily Pr-1 revealed up to 4.5% sequence variability in gorilla compared with the human sequence [105]. Members of the Pr-1 subfamily are absent from the chimpanzee genome either because the sequence has been lost after the divergence of gorilla and chimpanzee from the common ancestor or because the subfamily emerged independently in the gorilla and human genomes. In great apes, nucleotide variation of satellite repeats was investigated using short- and long-read sequencing data, and repeat densities were measured and compared among ape species. Interestingly, satellite density varied considerably among ape species. At a certain level of abundance of the satellites in the genome, the satellite variants can become dominant, and subsequently result in intrachromosomal homogenization in species and formation of chromosome-specific arrays (Figure 5). Recently, chromosome-specific variation of AS repeats in human populations has been revealed [62,124,225]. Three AS sequences, namely D17Z1, D17Z1-B, and D17Z1-C which are localized adjacent to the centromere of human chromosome 17, show high variations [124]. These satDNA repeats have been widely investigated in a number of recent studies [226,227,228,229]. Each of the three aforementioned satellites can serve as a functional centromere to recruit CENP-A histone proteins; therefore, the functional multiple AS sequences located on a single chromosome are termed epialleles [230]. Approximately 70% of the analyzed population harbors assembled centromeres at the 16mer D17Z1 position, whereas the remaining 30% displays differential assemblies of centromeres at the D17Z1 and 14mer D17Z1-B loci. Centromere assembly is supported by the D17Z1-B epiallele in human artificial chromosomes, but to date no human individual with this allele has been detected. Therefore, it is speculated that individuals homozygous for the D17Z1-B epiallele may represent rare but viable variants in the human population. Likewise, human sex chromosomes have been explored by NGS analysis and several satellite sequences with X- and Y-linked variants that differ in size have been detected [62]. Ongoing research by several groups has built upon this foundation to understand satellite repeat variation [231,232], and further indicates that HOR variation in the centromeres might not be specific to human chromosome 17.

Over the course of the past two decades, AS sequence divergence has been the main focus of studies of satellites in primates. Array patterns of AS sequences in African green monkeys were identified with 1–5% divergence among inter- and intrachromosomal monomers [233,234]. Other primate genomes possess repeated units with 30–50% divergence in monomers among species, although the complete array exhibits only 1–10% variability [98,235,236,237,238,239]. Alexandrov et al. [100] reviewed and characterized the divergence and ancestry of AS in lower primates. The ancestral AS monomer, termed S1, may have acted as a source sequence to give rise to a S1–S2 dimer repeat, which is a typical sequence of Old World monkey genomes. This S1–S2 dimer is formed by combination of S1 with its S2 variant, followed by duplication during the evolution of lower primates. Conversely, the S3–S4 dimer, which is characteristic of New World monkeys, evolved by amplification of S1-like variants with different divergence rates. In certain species of the New World monkey genera *Pithecia* and *Chiropotes*, a trimer unit (combination of the S3, S4, and S5 monomers) is formed as a result of unequal crossing over between S3–S4 dimer repeats. The S5 monomer in this trimer unit evolved from S3 and S4, and shares their sequences. Each of the aforementioned HORs is located on several chromosomes forming the bulk of heterochromatin in the corresponding primate species. In some Old World monkey species, such as *Macaca fuscata*, the certain repeat units are almost identical with those of *Chiropotes* and *Pithecia*, including S1–S2 dimer repeats that remain conserved throughout the genera *Papio* and *Macaca* [100]. Some primates, such as great apes, have acquired novel monomers that have been duplicated and intermixed with older monomers [240,241,242]. As a result, three new suprachromosomal families (SF1, SF2, and SF3) emerged in great ape genomes. In humans, the new AS families are clustered in the centromeres of all autosomes but are absent on the Y chromosome, whereas in other apes they are distributed on almost every chromosome. Each satellite family represents a distinct chromosome-specific structure that is defined by 2–30 monomeric HORs arranged tandemly with more than 95% identity [240]. Each family can comprise thousands of copies of HOR arrays that can span 250–5000 kb sequences (reviewed in [125]). Certain AS families in humans that evolved into the new SFs can contain both ancestral and new monomers. These ancestral sequences might have amplified and accumulated mutations during their insertion or relocation into nonhomologous centromeres and other chromosomes. Owing to subsequent amplification of mutated sequences, a novel HOR could have been formed that now contains divergent copies of ancestor repeats eventually forming a new array [97,242].

Mutations (polymorphism) can occur in primate satellite sequences at both single bases, i.e., single-nucleotide polymorphism (SNP), or multiple nucleotides (structural variants), which may involve segments of HORs [243]. As described earlier, several cases have been reported in primates that show sequence variation in HORs to be species-specific, population-specific, or even chromosome-specific with different divergence rates. Most of these variations have been studied in AS repeats of humans [50], although divergence repeats have also recently been investigated in several NHPs using modern sequencing techniques [48,244]. However, these studies tend to focus on interspecific comparison, and population variability of satDNA sequences remains incompletely understood in NHPs. Research on satDNA sequence divergence or variation may provide further interesting genomic insights into the evolutionary processes among lineages or populations. Analysis of genome sequences has resulted in the characterization of a 187 bp megasatellite sequence named OwlRep, which is distributed in the heterochromatin of simian primates (infraorder Simiiformes) and owl monkeys [245]. Interestingly, primate infraorder Tarsiformes and suborder Strepsirrhini have only one copy of HSAT6, whereas several species of infraorder Simiiformes can carry many copies. Comparative sequence analysis of these copies revealed duplication of HSAT6 in New World monkeys and demonstrated that OwlRep probably originated during the divergence of owl monkey lineages from New World monkeys. In addition, species-specific variation in AS monomer size is reported among New World monkeys (sequence length from 340 to 350 bp) and hominids (171 bp). The CENP-B box was identified in the genome of three New World monkey species, including squirrel monkey (*Saimiri sciureus*), tamarin (*Saguinus oedipus*), and marmoset (*Callithrix jacchus*) [244]. The CENP-B sequence of each species not only varied in length but was also located at different chromosomal positions.

## 7. Evolutionary Birth and Expansion of Satellite DNA

As described earlier, satDNA repeats are highly variable sequences that represent species- or genus-specific genomic fractions and reflect trajectories of short-term evolutionary changes [21,246,247,248]. Nevertheless, the significance of these sequences in studying genomic functions and structuring during different evolutionary events, together with increasing data on their functional role have been widely documented. However, their evolutionary dynamics and origin are still poorly understood. A general assumption is that intraspecific variation in a monomer of different satellite families can be fixed permanently in the genome [87]. Phylogenetically close lineages share common satellite sequences derived from an ancestral genome. Any differential copy number or acquired polymorphism within this sequence as a representative of a distant lineage can result in interspecific divergence [201,225,249,250]. Simultaneous occurrence of intraspecific homogenization of particular satellite repeat groups and fixation of species-specific mutations, as well as satellite conversion, all contribute to formation of a new species-specific satellite sequence. This phenomenon can have an important impact on driving molecular-level speciation [36]. Although the basic evolutionary mechanism that causes molecular organization and diversity is unknown, a common perception is that concerted evolution can result in unequal relocation of satellite repeats within the same or different chromosomes through various mechanisms, such as unequal crossing over, rolling-circular replication, gene conversion, and TE-mediated transfer [90,251]. Such events may subsequently trigger new means of amplification, thus giving rise to the formation of novel arrays of satDNA [62,127,228,232]. Certain chromosome-specific human AS groups have been highlighted with a specific age gradient for each chromosomal locus, which suggests that new AS repeats expanded in centromeric regions during evolution [29,180,229]. Data on AS sequence evolution in NHP species are rare, but several recent papers explore the mechanisms of evolution of primate satellites.

Ruiz-Ruano et al. [252] proposed a hypothesis stating that satellite repeats arise through de novo duplication of a genomic segment. As a result, a novel tandem repeat is formed at a different genomic position. Several possible mechanisms may facilitate this phenomenon, such as single-strand slippage during DNA replication or reinsertion of replicated copies from extrachromosomal DNA. The newly born satellite repeat can be dispersed throughout the genome by different processes, such as transposition or insertion of replicated copies of extrachromosomal DNA. This can eventually lead to amplification of certain loci and expansion in sequence size of the satellite repeat mostly by unequal crossing over. According to this evolutionary model, a tandem monomer of 15 bp or less can arise in several genomic loci randomly; therefore, both microsatellite and short minisatellite repeats may arise via this evolutionary process. Short repeated monomers can subsequently form long arrays by various continuously occurring processes of divergence and duplication [253,254]. An example of such arrays is HORs, which might be evolutionary products of homogenization and duplication of shorter tandem units [87]. The above-mentioned hypothesis proposed by Ruiz-Ruano et al. [252] can be integrated with another proposal, termed the “Library hypothesis”, which offers insights into the distribution of satDNA families among lineages [87,252,255,256,257,258]. As described in this section, species that are phylogenetically close share a common set of preserved satellite sequences derived from their ancestor, and each sequence shows differential amplification among each species.

According to the Library hypothesis, the main driving forces of satellite repeat dissemination are transposition and rolling circle replication. Although no evidence has been reported that either of these mechanisms drives the spread of satDNA in the genome, the expansion of satellites has been suggested to occur through a rolling circular process [259,260]. There is increasing evidence to highlight that TEs can mediate the emergence of satellite sequences and their mobilization in the genome [258,261]. Transposable elements can be important contributors in shaping satDNA evolution by playing a crucial role in the formation of a new library of satellite repeats, their dispersion in the genome, and even their amplification into longer arrays in certain cases [258]. It is now considered that the origin and dissemination of new satellites mediated by transposition has been underestimated and there may be more cases of TEs and satDNA evolutionary association [262]. After the birth and dissemination of a satDNA family within a genome, each satellite sequence might either evolve independently and freely through sequence divergence or follow concerted evolution. During this phase, all tandem repeated units may show cohesive evolution [263]. In contrast to what would likely happen in the absence of selective pressure, different units of a satDNA family could show a higher degree of intraspecific resemblance and interspecific divergence by following a concerted evolutionary process [36,87]. Gradual concerted evolution can occur in a step-wise manner through a specialized mechanism termed “molecular drive” [264,265]. This model proposes that new satDNA variants, formed through accumulation of mutations in the monomers, are distributed throughout the array by different mechanisms such as transposition, unequal crossing over, or reinsertion of extrachromosomal replicates (Figure 5). All these evolutionary events, together with gene conversion, can result in homogenization of evolved satellite sequences. These variants can then undergo fixation in the population by sexual reproduction. An important, interesting aspect of concerted evolution is the variable degree of population differentiation. Concerted evolution of satellite repeat sequences has been observed among populations in different species [227,250,263,266], which supports the hypothesis that satDNA might serve as the main driving force of population-level genomic variability.

## 8. Enlightening the Dark Matter of the Genome: Modern Approaches and Challenges in Detecting satDNA Repeats

**Figure 6:**
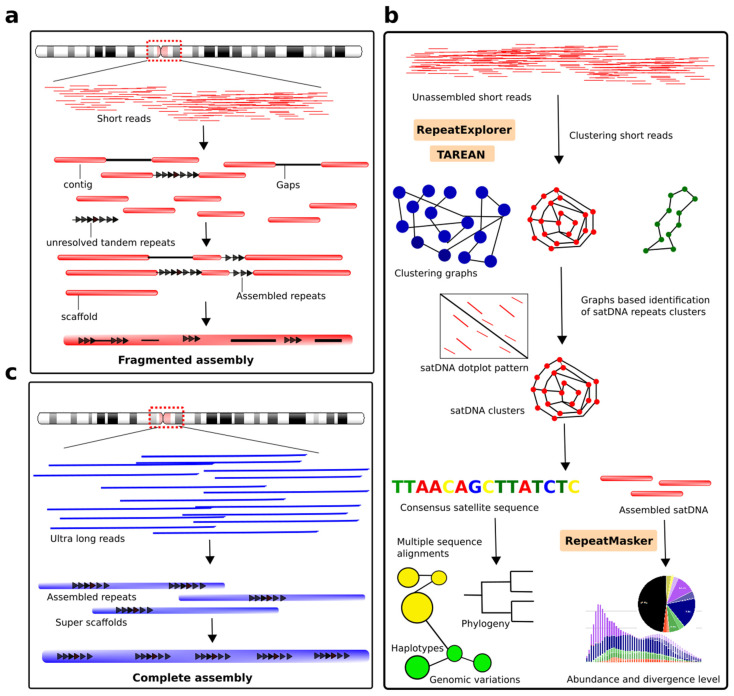
Schematic illustration of genome assembly limitations and new next-generation sequencing approaches for satellite DNA analysis. (**a**) Centromere region of the human chromosome that could not be assembled using short-read sequencing and is not represented in primary human assemblies. The assembly algorithms cannot be used for short reads (red lines) of the centromere owing to high-level reiteration of tandem repeats (black triangles) and therefore could not be recovered in the assembly. The fragmented assembly may contain gaps, thereby missing satellite DNA sequences causing bias to genome annotation. (**b**) A cheap alternative to analyze the satellite directly is the development of clustering-based pipelines, which can graphically predict different repeats and cluster them into groups. These programs yield assembled contigs, which can be further utilized for downstream analyses, such as repeats abundance, divergence, and comparative genomic analysis. (**c**) Recent developments (ultra-long-read technology) have successfully recovered the complete human genome assembly [279].

Par-genome assemblies of model and non-model primate species are currently being produced at an impressive rate, with unprecedented quality and contiguity by consortia and individual laboratories [267]. However, difficulties in assembling repeat-rich regions (genomic “dark matter”) limits insights into the evolution of genome structure and regulatory networks [19]. The largest assembly gaps remain in centromeric regions and acrocentric short arms, sites known to contain megabase-sized arrays of satDNA [225]. Complex repeat structures have very important evolutionary and biomedical functions. Therefore, the successful assembly of these repeats completely and accurately is paramount to obtain maximum implications of primate genomes. The extremely repetitive nature of satDNA sequences has presented a challenging task to generate de novo genome assemblies. High levels of variation in the abundance of these tandem repeats among species cause complications in both assembly procedure and algorithms [44]. Ideally, a high-quality genome assembly must represent accurately annotated features such as genes and complete repeated contents with correct chromosomal/scaffold positions. The assembly can be further utilized in new experiments to provide a better understanding of gene or repeat function and expression, for instance during investigation of differential expression among diverse conditions using RNA sequencing [268]. Significant variation in the natural abundance of tandem repeats exists in different organisms. This complicates assembly procedures and prevents development of algorithms that perform reliably in all cases. The first-ever human repeatome database to include tandem repeats was introduced in 1992 [269] and is now developed as “Repbase” [270]. Subsequently, NGS has revolutionized the development of modern resources, including new approaches for detection of repeats and availability of repeat databases. More than 50 bioinformatics tools have been developed to detect tandem repeats, while numerous publicly accessible databases have been established containing an enormous amount of data applicable in various fields, such as medicine, agriculture, and forensics. Some well-known databases are the Human Genome Browser by UCSC [271], the Tandem Repeats Database (TRDB; [272], Dfam [273], and STRBase [274]. Most databases are based on data generated by well-known bioinformatic tools, e.g., “Tandem Repeats Finder” (TRF) [275] or “RepeatMasker” [52], which are widely used for the characterization of repeats. In particular, the RepeatMasker program is the preferred tool for repeat masking and identification in assembled genomes. However, the use of these two tools might not be sufficient to detect all repeats precisely because of their conservative approach, statistical errors, and lower prediction power in finding diverged repeats. To address these shortcomings, adequate statistical methodology integrated with a meta approach is required for accurate annotation of repeats. For instance, a program called “Tandem Repeat Annotation Library” (TRAL) [276] can considerably improve repeats annotation, and further identify repeat regions that might be under selection. The repeat detectors, as described above, can be employed to annotate repeat contents including satDNA; however, these programs require a longer sequence (contigs) as input that can be sourced from genome assemblies. These assemblies are extremely under-represented for satDNA and can miss certain satellite sequences altogether (Figure 6a). This problem can be highly challenging in primate genome annotation because long reiterative arrays of HORs are hard to stitch together, particularly when they are composed of multiple mega-base sequences. Therefore, HORs and AS repeats were missing, and centromeric regions were recognized as assembly gaps in previous assembly versions of the human genome [55]. Graphical-reference models of HOR arrays were then constructed from whole-genome sequences to configure the HOR monomers and fill these gaps [55]. However, these models were not able to place the HOR units on chromosomes in a linear fashion nor could they combine the long-ranged units into a complete array. More recently, a highly contiguous human assembly was published using Nanopore ultra-long sequencing [277]. This assembly successfully stitched the linear array of AS sequences and the DYZ3 satellites that span the regions between the long and short arm of the Y chromosome [277]. This improvement in length of ultra-long Nanopore reads up to 1 Mb attracted global attention and enabled the development of new techniques to achieve assembly of AS arrays of other primates, making up the most abundant portion of their satellitomes (Figure 6b). It would be worthwhile to enhance the efficiency of detection of AS variants and their changes of copy number among different populations, species, or genera. The recent accomplishment of end-to-end assembly of the entire X chromosome of humans has further revolutionized genome science and opened the way to enable the detection of multi-megabase satellite arrays in the pericentromeric regions [8]. High coverage of ultra-long-read sequencing (PacBio and Oxford Nanopore) integrated with complementary technologies, such as Hi-C and BioNano, and development of additional methods are required to achieve gapless genomes and resolve the long stretches of centromeric satellites. However, generation of an accurate assembly is also a laborious and time-consuming task and requires deep sequencing, which can be costly, and in where the reference genome is unavailable, the approach will not be useful for characterizing satellite repeats. This could be a further challenge, especially when the goal of the study is to predict novel repeats. A similarity-based clustering algorithm has been developed that evaluates all sequence comparisons between unassembled reads and groups the repeated sequences into clusters [278] (Figure 6c). Two efficient pipelines based on this method are RepeatExplorer [279] and Tandem Repeat Analyzer (TAREAN) [280]. These tools have proved to be the most successful approaches to date, particularly in the characterization and quantification of satellite repeats.

## 9. Concluding Remarks

Satellite DNA is among the most fascinating components of the primate genome. Satellite repeats are extremely variable in sequence length. They have evolved rapidly and serve as vital sources for genetic divergence. Here, we have discussed the different aspects of satellite DNA evolutionary paradigms, genomic organization, diversity, and functional significance. We have reviewed different kinds of satDNA sequences that have been characterized in primate genomes, and outlined their proportion and structure as well as variation among primate lineages. These sequences are involved in different fundamental functions and are critically important in driving karyotypic evolution. A remarkable feature of these elements is their intense degree of interspecific and intraspecific variation and divergence, which might be important factors in primate speciation. We have further reviewed hypotheses on the evolutionary origin, genomic birth, and expansion of satDNA. Presently, certain technological limitations hamper the progress of in-depth and precise analysis, which demands the development of efficient computational tools and sequencing technologies for complete assembly of the centromeric and telomeric regions to overcome these limitations and boost further studies. Significant efforts are required from the research community to elucidate these genomic regions, which have been ignored for too long.

## Supplementary Materials

The following are available online at https://www.mdpi.com/2073-4409/9/12/2714/s1, Table S1: SatDNA repeats described in this review.
