# Supplementary material for: Dark Matter of Primate Genomes: Satellite DNA Repeats and Their Evolutionary Dynamics
Source: Cells. 2020 Dec 18;9(12):2714. doi: 10.3390/cells9122714 (PMC7767330; doi:10.3390/cells9122714)
Supplement: Supplementary file 1 [file cells-09-02714-s001.pdf]

Supplementary Table S1. SatDNA repeats described in this review.

Satellite repeat	Sub-type	Primate species	Chromosomal location	Monomer Size	Repeat array length	Reference
HSAT	HSAT1	*Homo sapiens, Cercopithecus pogonias, Cercopithecus solatus,*	Centromere	24 bp	566 bp	[181]
	HSAT2		Pericentromere	24 bp	1.77 kb	[223]
	HSAT3		Pericentromere	24 bp	3.6 kb	
	HSAT6		Centromere	-	126 bp	[132]
Non-alphoid satDNA	SatIII	*H. sapiens, Pan troglodytes, Gorilla beringei, Pongo pygmaeus*	Pericentromere	-	156 bp upto - 700 bp	[148,149]
OwlRep	OwlAlp1	*Aotus azarae*	Centromere	184 bp	-	[22,23]
	OwlAlp2		Pericentromere	344 bp	-	[22,23]
Beta satellite	D4Z4	*H. sapiens, Macaca mulatta, Macaca nemestrina, Saimiri sciureus, Chlorocebus sabaeus, Ateles geoffroyi*	Acrocentre loci, (10q26 and 4q35)	3.3-kb	several kb	[147]
Microsatellites	AP74	*H. sapiens, P. troglodytes, Ateles chamek, Ateles belzebuth, Alouatta caraya, Lemur catta*	Telomere/Sub-Telomere	126 and 176 bp	-	[76,77]
	MsH42	*H. sapiens*	q25.1	10 to 100 bp	863 bp	[77]
AS repeat	Cap-A	*Cebus apella*	Telomere	-	1500 bp	[161]
	Cap-B			-	342 bp	
Higher-order repeats	MarmoSAT	*Callithrix jacchus, Callimico goeldii*	Subtelomere	171 bp	338 bp	[157]
Sex-linked satDNA	Gamma satellite	*H. sapiens*	Pericentromere	3, 9, or 24 kb	220 bp	[27,179]

Note: The dash "-" denotes the data for this repeat type is unknown.
